# Geographical Accessibility Does Not Affect Prognosis After Living‐Donor Liver Transplantation

**DOI:** 10.1002/ags3.70099

**Published:** 2025-09-27

**Authors:** Kyohei Yugawa, Takeo Toshima, Shinji Itoh, Tomoharu Yoshizumi

**Affiliations:** ^1^ Department of Surgery and Science Graduate School of Medical Sciences, Kyushu University Fukuoka Japan

1

Matsushima et al. concluded that living‐donor liver transplantation (LDLT) patients residing in remote areas might experience delays in re‐transplantation or emergency treatment, resulting in a negative impact on survival rates with analysis using 301 LDLT cases because the number of facilities capable of performing LDLT limited in Japan [1]. In their study, patients were divided into three groups based on the distance from their homes to the transplant center: Group 1, 0–9 miles; Group 2, 10–40 miles; and Group 3, > 40 miles. The greater distance (Group 3) was independently associated with LDLT patient poor survival. Regarding post‐PTLD complications, the incidence of hepatic artery thrombosis was higher in Group 3 than other groups.

We would like to share our results on the association between distance and recipients' survival at Kyushu University Hospital. Our cohort included 587 adult recipients who underwent LDLT at our transplant center between January 2008 and February 2024. Patients were classified into three groups based on the same criteria: Group 1 (*n* = 118, 20.1%), Group 2 (*n* = 172, 29.3%), and Group 3 (*n* = 297, 50.6%). We found no significant difference in 1‐ and 5‐year graft survival (95.7% vs. 91.7% vs. 88.6%; 88.9% vs. 85.0% vs. 81.8%, *p* = 0.241) (Figure 1). Additionally, there were no significant differences in the incidence of rejection or de novo malignancy among the three groups.

**FIGURE 1:**
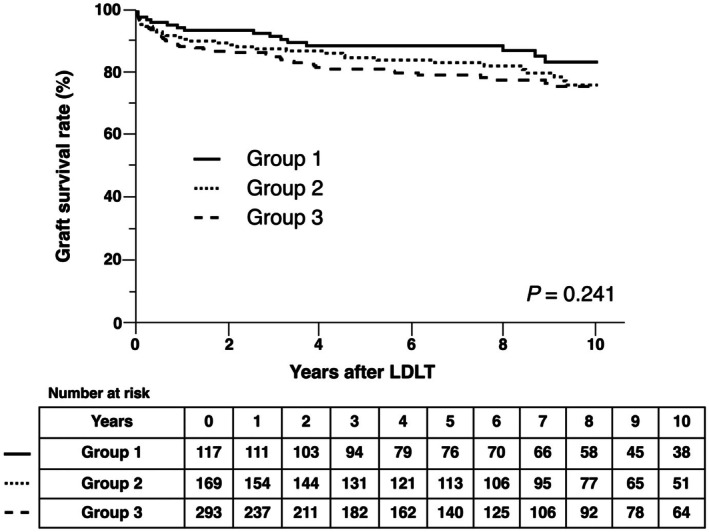
Kaplan–Meier curves of graft survival according to distance to our transplant center. There was no significant difference among the three groups (Log‐rank, *p* = 0.241).

Consistent with a recent report [2], our findings suggest that medical access to liver transplantation—as well as distance alone—does not affect long‐term outcomes. One possible explanation is the relatively easy access to transportation in our region, with excellent access to public transportation such as highways, trains, and airports, regardless of distance. While their research has revealed the relationship between transplant prognosis and simply geographic distance, it is more important to consider accessibility in a broader sense. Second, our facility has established a system to provide continuous and specialized post‐LDLT care for patients from other prefectures. Specifically, we coordinate closely with base hospitals in each region to provide regular follow‐up care by experienced hepatologists. Given the current shortage of transplant internists in Japan, this is extremely meaningful in terms of training transplant specialists by providing guidance on how to manage long‐term complications after LDLT or immunosuppressive agents. Finally, in neighboring prefectures, transplant surgeons regularly visit twice a month or more to directly follow‐up LDLT patients in those regions. These efforts minimize regional disparities in post‐transplant care and enable the provision of continuous, high‐quality post‐LDLT management to patients living in remote areas.

LDLT continues to expand in Japan for patients with end‐stage liver disease who have access to a suitable donor [3]. As transplant indications broaden, the number of recipients is expected to increase in the near future. In parallel, post‐transplant follow‐up, which involves complex care, will require more multidisciplinary support. Nationwide, it is essential to establish a medical environment that enables patients living in remote areas to lead their lives with confidence after surgery.

## Author Contributions

Study conception: K.Y., T.T., S.I., and T.Y. Study design: K.Y., T.T., S.I., and T.Y. Data acquisition: K.Y., T.T., and T.Y. Data analysis and interpretation: K.Y., S.I., and T.Y. Statistical analysis: K.Y. and T.T. Manuscript preparation: K.Y. and T.T. Manuscript editing: K.Y., T.T., S.I., and T.Y. Manuscript review: All authors.

## Disclosure

*Approval of the Research Protocol*: N/A.

*Registry and the Registration No. of the Study/Trial*: N/A.

*Animal Studies*: N/A.

## Consent

The authors have nothing to report.

## Conflicts of Interest

The authors declare no conflicts of interest relevant to this article, except that author Tomoharu Yoshizumi serves as an editorial member of *Annals Gastroenterological Surgery*.

## Linked Articles

This article is linked to Matsushima et al. paper. To view this article, visit https://doi.org/10.1002/ags3.70051.
